# The CzcCBA Efflux System Requires the CadA P-Type ATPase for Timely Expression Upon Zinc Excess in *Pseudomonas aeruginosa*

**DOI:** 10.3389/fmicb.2020.00911

**Published:** 2020-05-15

**Authors:** Verena Ducret, Manuel R. Gonzalez, Sara Leoni, Martina Valentini, Karl Perron

**Affiliations:** ^1^Microbiology Unit, Department of Botany and Plant Biology, University of Geneva, Geneva, Switzerland; ^2^Department of Microbiology and Molecular Medicine, CMU, Faculty of Medicine, University of Geneva, Geneva, Switzerland; ^3^Institute of Pharmaceutical Sciences of Western Switzerland, University of Geneva, Geneva, Switzerland

**Keywords:** zinc, homeostasis, carbapenem, export, CadA, CzcCBA, resistance

## Abstract

Zinc (Zn) is a trace element essential for life but can be toxic if present in excess. While cells have import systems to guarantee a vital Zn intracellular concentration, they also rely on export systems to avoid lethal Zn overload. In particular, the opportunistic pathogen *Pseudomonas aeruginosa* possesses four Zn export systems: CadA, CzcCBA, CzcD, and YiiP. In this work, we compare the importance for bacterial survival of each export system at high Zn concentrations. We show that the P-type ATPase CadA, and the efflux pump CzcCBA are the main efflux systems affecting the bacterium tolerance to Zn. In addition, *cadA* and *czcCBA* genes expression kinetics revealed a hierarchical organization and interdependence. In the presence of high Zn concentrations, *cadA* expression is very rapidly induced (<1 min), while *czcCBA* expression occurs subsequently (>15 min). Our present data show that the fast responsiveness of *cadA* to Zn excess is due to its transcriptional activator, CadR, which is constitutively present on its promoter and promptly activating *cadA* gene expression upon Zn binding. Moreover, we showed that CadA is essential for a timely induction of the CzcCBA efflux system. Finally, we observed an induction of *cadA* and *czcCBA* efflux systems upon phagocytosis of *P. aeruginosa* by macrophages, in which a toxic metal boost is discharged into the phagolysosome to intoxicate microbes. Importantly, we demonstrated that the regulatory link between induction of the CzcCBA system and the repression of the OprD porin responsible for carbapenem antibiotic resistance, is maintained in the macrophage environment.

## Introduction

Living cells require trace-metals, such as Zn, Fe, Cu, Co, Mg, Mo, and Ni as co-factors for enzymatic reactions, protein folding, and regulatory actions (Andreini et al., 2008). They therefore play essential roles in all living organisms. However, excess of metals causes toxic effects by interacting with proteins and cells components or causing ROS production (Chandrangsu et al., 2017; Kim et al., 2019). Metal homeostasis, also called metallostasis, is therefore of high priority for all biological systems. As opposed to eukaryotes, bacteria lack of storage compartments they have evolved several metallostasis mechanisms allowing them to thrive under conditions of metal limitation or excess (Chandrangsu et al., 2017). This is, in particular, the case of Zn, one of the most abundant metals in living organisms, including bacteria, and a major cofactor involved in enzyme function (Outten and O’Halloran, 2001; Andreini et al., 2008). During mammals infection, Zn is sequestered by the host cells by a defense strategy called nutritional immunity, rendering the metal poorly accessible to bacterial pathogens (Kehl-Fie and Skaar, 2010). During this process, Zn is chelated by various proteins such as calprotectin secreted at the site of infection by neutrophils (Corbin et al., 2008), or psoriasin on the skin (Glaser et al., 2005), that ultimately inhibit pathogen proliferation. Conversely, when bacteria are taken up by phagocytic cells such as macrophages or protozoa, they must contend with a strong boost of Zn and Cu, discharged into the phagosome by metal-containing vesicles (Ong et al., 2014; Djoko et al., 2015; Hao et al., 2016). Thus, to ensure successful infection, bacteria must first deal with a phase of Zn starvation, rapidly followed by a toxic condition of Zn excess.

*Pseudomonas aeruginosa* is an opportunistic Gram-negative bacterium that possesses several tools to control Zn homeostasis. Importantly, these systems contribute to the versatility, virulence and antibiotic resistance of this pathogen (reviewed in Gonzalez et al., 2019).

Four Zn export systems have been described in *P. aeruginosa* (Gonzalez et al., 2019). The most characterized is the CzcCBA efflux pump (Hassan et al., 1999; Perron et al., 2004), which is homologous to the system found in the metal-resistant bacterium *Cupriavidus metallidurans* (Mergeay et al., 2003). This efflux pump belongs to the RND (Resistance-Nodulation-Division) group of the HME (Heavy Metal Efflux) family (Tseng et al., 1999), corresponding to the first layer of metal resistance in bacteria (Nies, 2003). It can expel excess Zn, Cd, and Co from the periplasm or cytoplasm, directly outside the bacterium (Nies, 1999). The expression of this efflux pump is regulated by the CzcRS two-component system (TCS) in which CzcS, the inner membrane-located sensor protein, is capable of detecting excess levels of periplasmic Zn (or Cd) and activates the CzcR protein by phosphorylation (Wang et al., 2017). The CzcR response regulator then acts as a transcriptional activator of *czcCBA* genes expression, leading to metal resistance. Simultaneously, CzcR activates its own transcription and represses the transcription of the *oprD* gene (Perron et al., 2004). OprD is a porin involved in the uptake of basic amino acids and small peptides and is also the route of entry of carbapenem antibiotics such as imipenem and meropenem (Trias and Nikaido, 1990a, b). Therefore, by repressing *oprD* transcription, CzcR leads to carbapenem resistance. This mechanism, linking metal and antibiotic resistance, is called “co-regulation” (Baker-Austin et al., 2006).

A second layer of metal resistance in bacteria is provided by the cation diffusion facilitator (CDF) transporters (Nies, 2003). CDFs are homodimers located in the cytoplasmic membrane and to allow the export of Zn and other metals from the cytoplasm to the periplasm by way of a proton gradient (Kolaj-Robin et al., 2015). Some data suggest that paralogs of CDFs may also form functional heterodimers (Uebe et al., 2011). *P. aeruginosa* possess two Zn CDFs, namely CzcD and YiiP that confer a slight Zn tolerance and have been shown to affect periplasmic Zn homeostasis as well as outer membrane integrity in *P. aeruginosa* (Salusso and Raimunda, 2017).

The third layer of metal resistance (according to Nies, 2003) is conferred by the P-type ATPase superfamily of transporters that use ATP as energy for the transport of metal through the cytoplasmic membrane. Found in all kingdoms of life, they are very efficient for the transport of ions or lipids across membranes, guaranteeing rapid cell homeostasis (Palmgren and Nissen, 2011). Classified in five distinct subfamilies according to their sequences (Axelsen and Palmgren, 1998), the P1B subgroup of P-type ATPases is involved in heavy-metal homeostasis. Although some of these systems might import divalent cations from the periplasm to the cytoplasm, the P-type ATPases involved in direct metal resistance are mainly export systems (Nies, 2003). P-type ATPases involved in the export of Cd, Zn, and Pb have previously been described in *Pseudomonas* species (Lee et al., 2001). This protein, called CadA (or ZntA), has been reported in different genera of Gram-positive and Gram-negative bacteria, either plasmid-borne or on the chromosome (Nucifora et al., 1989; Lebrun et al., 1994; Rensing et al., 1997; Maynaud et al., 2014). The regulation of CadA/ZntA expression is mediated by the CadR (or ZntR) transcriptional regulator that belongs to the MerR family of response regulators (Brown et al., 2003). Upon binding the metal, MerR-like proteins are capable of inducing transcription by modulating the conformation of the *cadA*/*zntA* promoter DNA (Outten et al., 1999). The gene encoding the *P. aeruginosa* CadR protein is located upstream of *cadA* and transcribed in the opposite direction. Its response to metals has been investigated using a transcriptional reporter in *Escherichia coli* and showed a strong specificity for Cd (Brocklehurst et al., 2003).

The aim of this work was to define the importance of the systems involved in resistance to toxic Zn concentrations and their expression dynamics. More specifically, we followed the induction of expression of the two major efflux systems, CadA and CzcCBA, when the bacterium passes from a Zn-depleted environment, similar to Zn nutritional immunity conditions, to a Zn excess situation, mimicking the metal boost inside the phagolysosome. We found that CadA was the first system to be induced, thanks to CadR already located on the promoter. The expression of *cadA* stimulates the rapid induction of the highly efficient CzcCBA efflux pump. Using the THP1 macrophage cell line, we confirmed that Zn export systems are significantly induced when the bacterium is phagocytosed. Importantly, linked to the expression of these systems, *oprD* transcription is repressed, leading to impermeability to carbapenem antibiotics and therefore to resistance. Altogether, these data show the complex interplay between Zn export systems and indicate their involvement in response to phagocytosis by immune cells.

## Materials and Methods

### Bacterial Strains and Culture Media

The bacterial strains/plasmids and oligonucleotides used in this study are listed in Supplementary Tables S1, S2, respectively. Initial growth conditions and pre-cultures deficient in Zn, were performed at 37°C in modified Luria-Bertani medium (M-LB) prepared as previously described (Ellison et al., 2013) with the following modifications: LB medium (Miller supplier, Axon Lab) was depleted for divalent cations with a chelating resin (Chelex 100 sodium form, Sigma-Aldrich) at a concentration of 400 mg/L. For culture, except for maximum tolerable concentration (MTC) experiments, N,N,N′,N′-tetrakis(2-pyridylmethyl)-ethylenediamine (TPEN; Brunschwig) was added to the medium at a final concentration of 30 μM to further remove any remaining traces of zinc. Glassware needed for the experiments was treated with the resin Chelex 100 (400 mg/L in water) for 1 h at room temperature before sterilization. When required, antibiotics were added to the media at the following concentrations: 200 μg/mL carbenicillin (Cb), 50 μg/mL gentamicin (Gm), and 50 μg/mL tetracycline (Tc) for *P. aeruginosa* or 100 μg/mL ampicillin (Ap), 15 μg/mL Gm, and 15 μg/mL Tc for *E. coli*.

### Plasmid Construction and Gene Deletion Mutants

Chromosomal gene deletions were performed by homologous recombination. Two fragments of 400–600 bp flanking the gene of interest were amplified by PCR (primers listed on Supplementary Table S2) using *P. aeruginosa* genomic as template DNA. The two fragments were inserted into the suicide plasmid pME3087 using the Gibson Assembly Cloning Kit (New England Biolabs), or standard molecular procedures (Sambrook and Russell, 2001), transformed into *E. coli* DH5α strain by heat-shock for verification and amplification, then transformed into *P. aeruginosa* by electroporation and selected on Tc (Choi et al., 2006). Merodiploids were resolved as previously described (Ye et al., 1995) and the deletion was confirmed by PCR and sequencing.

*cadA::gfp* and *czcCBA::gfp* fusions were constructed from the pBBR1-*gfp* plasmid as follows: *cadA* or *czcCBA* promoter regions were amplified by PCR with primers 1198/1199 and 688/689, respectively, either from *P. aeruginosa* genomic DNA for wild type promoters, or from *in vitro* synthetized DNA (GeneArt, Thermo Fisher Scientific) for mutated promoters −2 and −IR. Fragments were then digested with KpnI and BglII enzymes, ligated into the pBBR1-*gfp* plasmid, transformed into *E. coli* DH5α, verified by sequencing and then transformed into *P. aeruginosa* as described before.

The plasmid pME6001 was used for complementation experiments. *cadA* or *cadR* genes and their respective promoter regions were amplified by PCR with primers 1205/1206 and 1188/1189, respectively, from *P. aeruginosa* genomic DNA and cloned into the pME6001 plasmid after digestion with BamH1 and HindIII restriction enzymes. Resulting plasmid was then transformed into *E. coli* DH5α, verified by sequencing and then transformed into *P. aeruginosa* as described before.

Maximum tolerable concentration. The MTC, corresponding to the highest concentration of metal allowing bacterial growth, was determined as follows: overnight cultures of wild type and mutants were diluted to an OD_600_ of 0.1 in M-LB medium, supplemented or not with an increasing concentration of Zn, as indicated in Table 1. Cultures were dispensed to 96-well plates for incubation at 37°C. After 24 h of growth, 0.2 mg/mL of 2-3-5-phenyl-2H-tetrazolium (INT, Sigma-Aldrich) was added to the cultures and incubated for 1 h at 37°C. The MTC was determined visually by observing the red coloration of INT reduced to formazan that appears in the event of bacterial growth (Eloff, 1998). A representative analysis of CMT is shown in Supplementary Figure S1. For MTC with complemented strains (Table 2), plasmids were maintained by adding gentamicin to the medium and the increasing concentrations of metals used are indicated in Supplementary Table S3.

**TABLE 1:** Maximum tolerable concentration (MTC) of Zn of the WT and export mutants.

	**WT**	**Δ*cadA***	**Δ*czcA***	**Δ*cadA*; Δ*czcA***	**Δ*czcD***	**Δ*yiip***	**Δ*czcD*; Δ*yiip***	**Δ*czcR***	**Δ*cadR***
MTC	8	5	3	3	8	8	8	3	6

*Maximum tolerable concentrations of ZnCl_2_ (in mM) after 24 h of growth at 37°C in LB liquid medium containing 0, 1, 2, 3, 4, 5, 6, 7, 8, 9, 10, 11 and 12 mM ZnCl_2_ as represented in Supplementary Figure S1. Values represent the mean of three independent determinations.*

**TABLE 2:** Effect of *cadA* or *cadR* deletion on MTC.

	**WT pME6001**	**Δ*cadA* pME6001**	**Δ*cadA* pME6001*-cadA***	**Δ*cadR* pME6001**	**Δ*cadR* pME6001*-cadR***
ZnCl_2_	8	5	8	6	8
CdCl_2_	4	0.25	4	1	4
CoCl_2_	1	1	1	1	1
NiCl_2_	2	2	2	2	2
CuCl_2_	4	4	4	4	4
Pb(NO_3_)_2_	10	8	10	8	10

*Maximum tolerable concentration of the WT, and of the Δ*cadA* and Δ*cadR* mutants carrying an empty plasmid, or a plasmid containing the mutated gene. MTCs are given in mM. Values represent the mean of three independent determinations. Metal concentrations in Supplementary Table S3.*

### Growth Experiments

For growth experiments, overnight cultures of wild type and mutant strains were diluted to an OD_600_ of 0.1 in M-LB medium, supplemented or not with zinc, transferred to 96-well plates and incubated at 37°C with shaking. Absorbance at 600 nm was monitored every 15 min using a Microplate reader (BioTek Instruments).

### GFP Fusion Assays

For GFP reporter experiments, overnight cultures of strains carrying the plasmid *cadA::gfp* or *czcCBA::gfp* were diluted to an OD_600_ of 0.1 in M-LB medium and incubated for 2.5 h. Cultures were then induced by adding the designated concentration of Zn. Absorbance at 600 nm and green fluorescence at 528 nm were monitored every 2.5 min using a Microplate reader (BioTek Instruments). Time “t0” indicated on the figures corresponds to the time of Zn addition to the cultures. The indicated arbitrary units correspond to the fluorescence values of the GFP normalized by cell density.

### Quantitative RT-PCR Analysis

Quantitative RT-PCR procedures were performed in duplicate starting from three independent experiments. Overnight cultures were diluted to an OD_600_ of 0.1 in M-LB and incubated for 3 h as described previously. 0.5 mL of each culture was added to 1 mL of RNA protect bacteria reagent (Qiagen) immediately prior to zinc addition (t0) and after several time points as indicated in Figures 2A, 3. Total RNA was extracted with RNeasy columns (Qiagen) according to the supplier’s instructions and treated with RQ1 RNase-free DNase (Promega) for 2 h at 37°C to remove residual DNA. After phenol/chloroform extraction, RNAs were precipitated and the pellets resuspended in 25 μl of RNase-free water. For cDNA synthesis, 500 ng of RNA was reverse-transcribed using random primers (Promega) and improm-II reverse transcriptase (Promega) according to the manufacturer’s instructions. Quantitative PCR was performed using Power SYBR Green PCR Master Mix (Thermo Fisher Scientific), according to the supplier’s instructions. Results were analyzed as previously described (Schmittgen and Livak, 2008) and normalized with the *rpoS* gene (PA3622). Primers used for qPCR are listed in Supplementary Table S2.

### CadR Expression and Purification

For CadR expression and purification, the *cadR* open reading frame was amplified by PCR using 1182 and 1185 primers and cloned into the BamHI site of the GST-fusion pGEX-2T vector yielding pGEX2T-*cadR* plasmid. This plasmid was verified by sequencing and transformed into *E. coli* BL21 DE3 strain for protein expression. A 100 mL culture of *E. coli* BL21 DE3 carrying the pGEX-2T-*cadR* was grown to an OD_600_ of 0.8. CadR expression was induced with 1mM Isopropyl β-D-1 thiogalactopyranoside (IPTG, Axon Lab) for 2 h at 37°C and cells were harvested by centrifugation. For purification, the bacterial pellet was resuspended in 4 mL PBS (Dulbecco’s Phosphate Buffered Saline, Sigma-Aldrich) containing protease inhibitors (cOmplete, EDTA-free, Roche), 1 mM DTT and 2mg/mL lysozyme. Cells were sonicated and centrifuged (10 min 13,000 rpm, 4°C). The supernatant was loaded onto a 0.6 mL glutathione-sepharose 4B (GE Healthcare) column (equilibrated with PBS, 1 mM DTT) and incubated for 6 h at room temperature on a rolling wheel. The column containing the GST-CadR protein was washed with 3 × 5 mL PBS-1 mM DTT. 1 mL PBS containing 1 mM DTT and 100 Units of Thrombin (GE Healthcare) was loaded onto the column and incubated overnight at room temperature on a rolling wheel. The flow-through was concentrated and dialyzed against storage buffer (PBS, 1 mM DTT, 50% glycerol) using Amicon Ultra-4 (Nanopore). Purity was verified on an SDS-PAGE 4–12% gel (Mini-PROTEAN Bio-rad) stained with Coomassie blue (Supplementary Figure S2; Sambrook and Russell, 2001). CadR purified protein was stored at −70°C until use.

### Electrophoretic Mobility Shift Assay

The ability of the CadR protein to bind the *cadA* promoter was characterized by electrophoretic mobility shift assay (EMSA). A 200 bp DNA fragment containing the WT or the modified (−2 and −IR) *cadA* promoter was amplified by PCR with primers 1111/1112 as described before for *cadA::gfp* fusion. The *czcD* promoter was amplified from genomic DNA with primers 1101/1102. Binding assays were carried out in binding buffer according to (Ellison et al., 2013), lacking zinc (10 mM Tris pH 8.0, 40 mM KCl, 10 mM MgCl_2_, 1 mM DTT, and 5% glycerol). Reaction mixtures were performed with or without 250 nM CadR protein and 25 ng DNA. A zinc gradient was achieved by adding EDTA or zinc to the amounts indicated in Figure 4B. Samples were then incubated at room temperature for 30 min and separated by electrophoresis at 4°C on a 7.5% polyacrylamide native gel containing 2.5% glycerol in Tris borate buffer. For DNA detection, the gel was stained with ethidium bromide and viewed under UV light.

### Primer Extension

In order to determine the start of transcription of the *cadA* gene, overnight cultures of PAO1 WT strain were diluted to an OD_600_ of 0.1 and incubated for 2 h 30 min in M-LB. Cultures were then induced (+Zn) or not (−Zn) with 2 mM Zn for 15 min and total RNAs were extracted as described previously. Four microgram of DNAse-treated RNAs were reverse transcribed with the specific primer 1197 labeled with 5′Fluorescein (6-FAM) and the Improm-II reverse transcriptase (Promega) according to the manufacturer’s instructions. Plasmid p*cadA*-*gfp* was sequenced with the labeled primer 1197-FAM using the Thermo Sequenase Dye kit (Thermo Fisher Scientific) according to the manufacturer’s instructions. All fragment analysis was performed by capillary sequencing (Microsynth AG, Switzerland) using ILS600 as a size standard. All peaks were analyzed using PeakScanner2 software (Thermo Fisher Scientific).

### Footprinting Assay

DNase I footprinting assay was performed according to (Baraquet and Harwood, 2017). The *cadA* promoter was amplified on the p*cadA*-*gfp* plasmid by PCR using primers 1111/1197-FAM or 1112/1196-FAM in order to have both DNA strands labeled independently. 50 ng of gel-purified labeled promoters was mixed with or without 250 nM of CadR purified protein in 40 μl of EMSA binding buffer (see above). The reaction was incubated for 30 min at room temperature. MgCl_2_ and DNAses I (RQ1, Promega), were added to the reaction and incubated for 2 min at room temperature. The reaction was stopped by quickly adding 60 μl ddH2O and 100 μl phenol/chloroform/IAA then vortexing. Eighty microgram of the aqueous phase containing digested DNA was EtOH precipitated with 8 μl 3M sodium acetate and 1 μl Glycogen (Roche). The pellet was resuspended in 12 μl water. Fragment size analysis and sequencing (with primers 1197-FAM or 1196-FAM) were performed as described for Primer extension analysis.

### Macrophage Experiments

Human monocytic THP-1 cells were cultivated at 37°C with 5% CO_2_ in RPMI 1640 medium (Gibco, A10491-01) supplemented with 10% heat-inactivated fetal bovine serum (FBS), 50 μM β-mercaptoethanol (Gibco), 100 U/mL Penicillin and 100 μg/mL Streptomycin (Pen Strep, Gibco). To differentiate THP-1 into macrophages, 100 ng/mL phorbol-12-myristate-13-acetate (PMA, Fluorochem) was added to 10^6^ THP-1 per mL. 20 mL was then dispensed to a 9 cm Petri dish and incubated for 72 h.

For the infection, the medium containing PMA was removed and substituted with 20 mL of the supplemented RPMI medium lacking antibiotics but containing *P. aeruginosa* at 2.5 × 10^6^ CFU/mL (multiplicity of infection of 2.5). After 1 h of infection, the supernatant was removed and replaced with fresh medium containing 200 μg/mL gentamicin in order to eliminate extracellular bacteria. To follow *P. aeruginosa* gene expression inside differentiated THP-1 cells, the infected macrophages were lysed with a mix of 2 volumes of RNA protect reagent (Qiagen) and 1 volume of 0.3% Triton X-100. Expression during phagocytosis was compared to the expression of extracellular bacteria grown for 1 h in the presence of macrophages. Total RNA and qRT-PCR were performed as described above.

## Results

### The P-Type ATPase CadA Affects Zn Resistance

In *P. aeruginosa*, four systems are known to participate in zinc export (Gonzalez et al., 2019): the CzcD and YiiP cation diffusion facilitators (CDFs), the CzcCBA Heavy metal Resistance Nodulation Division (HmRND) efflux pump and the CadA P-type ATPase (also called ZntA). In order to determine which system is the most important in resistance to high Zn concentrations, we evaluated the MTC of Zn for the WT and the individual mutants deleted for each of these systems (Table 1 and Supplementary Figure S1).

Under our experimental conditions, deletion of either the *yiiP* or *czcD* gene had no effect on Zn tolerance with an MTC of 8 mM, identical to the WT strain. Since the CDFs might complement each other (Salusso and Raimunda, 2017), we created a *yiiP*/*czcD* double deletion mutant. Again, no effect on Zn tolerance was observed.

**FIGURE 1:**
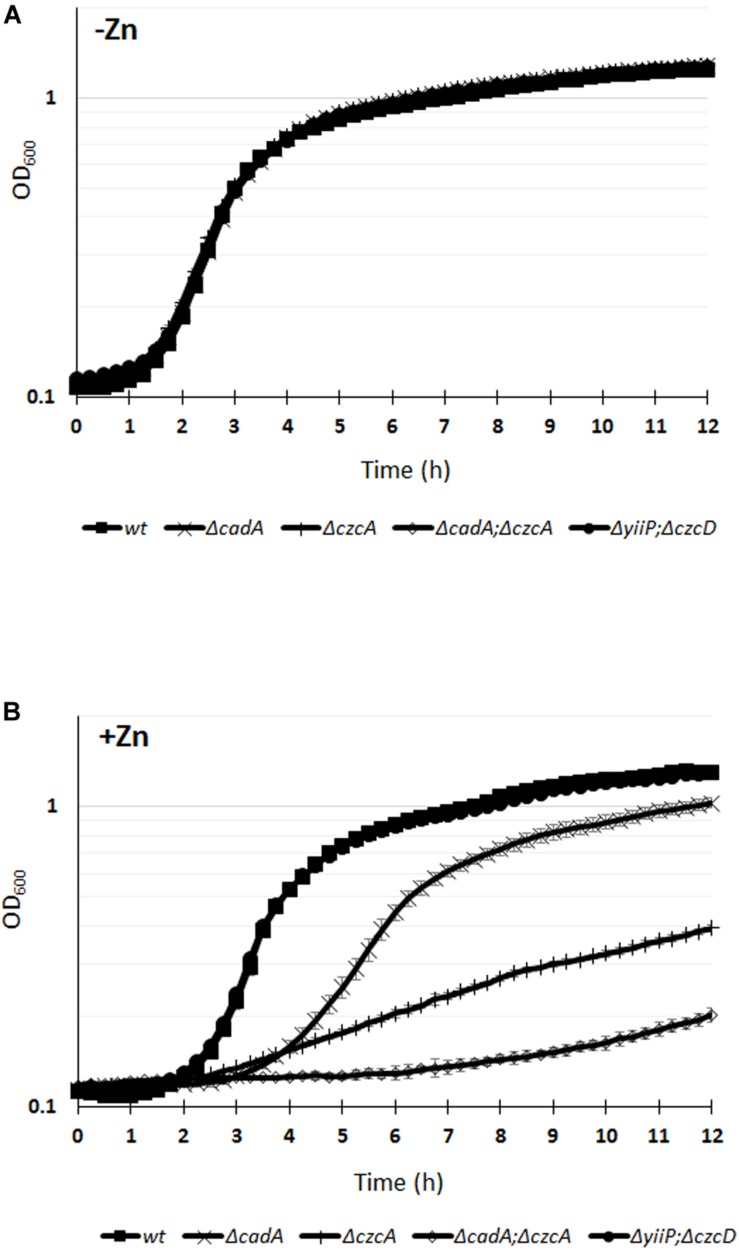
Growth curves of *P. aeruginosa* WT strain and of the various mutants cultivated in M-LB medium in the absence **(A)**, or presence **(B)** of 2 mM ZnCl_2_. Error bars represent the standard deviations of three measurements.

Deletion of the *czcA* gene, causing the inactivation of the CzcCBA efflux pump (Perron et al., 2004), or deletion of the *cadA* gene, encoding the P-type ATPase, showed an increased Zn-susceptible phenotype with MTCs values of 3 and 5 mM, respectively (Table 1). The *cadA/czcA* double mutant displayed a MTC of 3 mM Zn, similar to the *czcA* mutant. Considering that the MTC test reports on growth after 24 h, we decided to monitor the growth of the WT and the various mutants over a 12 h period in the absence or presence of 2 mM Zn (Figure 1). All the tested strains were unaffected in a medium lacking Zn excess (Figure 1A). While no susceptible phenotype was observed for the *yiiP*/*czcD* double mutant in the presence of 2 mM Zn, a delay in the growth kinetics was clearly visible for the Δ*cadA* and the Δ*czcA* mutant (Figure 1B). Interestingly, in the presence of Zn the growth of the *cadA/czcA* double deletion mutant was more strongly affected when compared to single deletion mutants (Figure 1B), suggesting a synergy between the two systems that could not be highlighted using the MTC test.

### Dynamics of Zn Resistance

**FIGURE 2:**
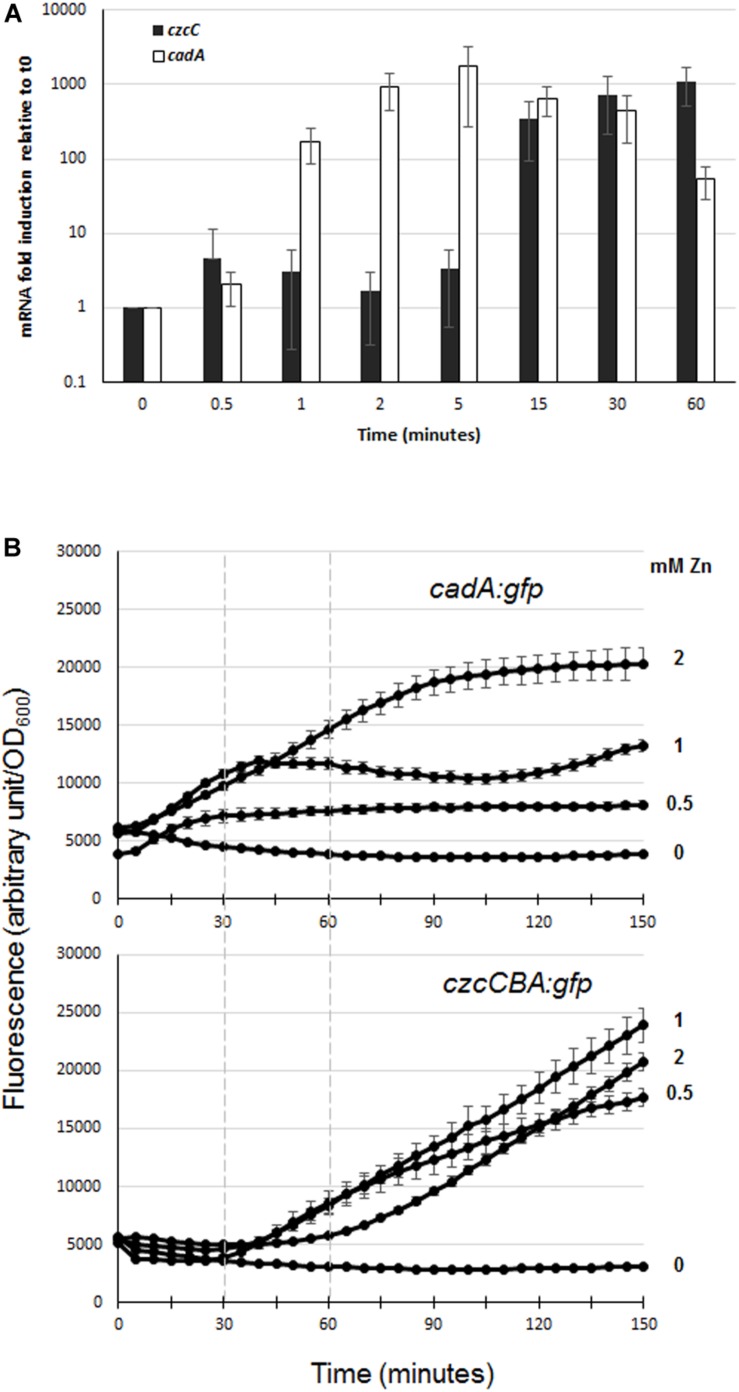
Expression of *czcC* and *cadA* export systems in the presence of Zn. **(A)** qRT-PCR analysis of *czcA* and *cadA* mRNAs after addition of 2 mM ZnCl_2_. The amount of mRNA is represented relative to time 0 (set to 1), before addition of ZnCl_2_. Results are normalized using the *rpoS* gene and standard deviations (error bars) of three independent experiments are indicated. **(B)** Fluorescence measurement of *cadA::gfp* (upper panel) and *czcA::gfp* (lower panel) after the addition of various concentrations of ZnCl_2_ (indicated on the right). Dashed lines on the graphs facilitated the comparison of fluorescence induction. Values are normalized by optical density (OD_600_). Standard deviations (error bars) of three measurements are indicated.

In order to evaluate the dynamics of zinc resistance in *P. aeruginosa*, we compared the induction kinetics of the two major export systems, CadA and CzcCBA, following zinc addition. To do so, we performed qRT-PCR analyses on RNAs extracted just before (time 0) and at different times after addition of 2 mM Zn to the culture medium (1, 5, 15, and 60 min; see Figure 2A). Already after 1 min, the amount of *cadA* mRNA had increased more than 100-fold, reaching the maximum of induction (1700-fold higher than at time 0) at 5 min and dropping to 70-fold at 1 h. The induction of *czcC* gene expression (the first gene of the *czcCBA* operon) was detected at 15 min and exceeded the level of *cadA* gene expression at 1 h. These data showed that CadA is the first system to be induced and to react to zinc excess.

To determine the different responsiveness of these two systems and the sensitivity of their Zn-dependent induction, we followed their induction after the addition of different concentrations of Zn (0, 0.5, 1, and 2 mM) using GFP reporter transcriptional fusion (Figure 2B). For this purpose, *czcCBA* and *cadA* promoters were fused to the green fluorescent protein gene (*gfp*) and these reporters were then transformed into a WT *P. aeruginosa* PAO1 strain. At all concentrations tested, we observed a very rapid induction of fluorescence with the *cadA::gfp* fusion, while a lag of approximately 30 min to 1 h was necessary to observe fluorescence controlled by the *czcCBA* promoter (Figure 2B). Interestingly, the fluorescence intensity of the *cadA::gfp* transcriptional fusion reached a plateau in all tested conditions, the maximal level of expression being determined by Zn concentration; while the *czcA::gfp* transcriptional fusion expression increased steadily during the 2.5 h of the analysis.

### CadR Is the Transcriptional Activator of *cadA* in the Presence of Zn Excess

**FIGURE 3:**
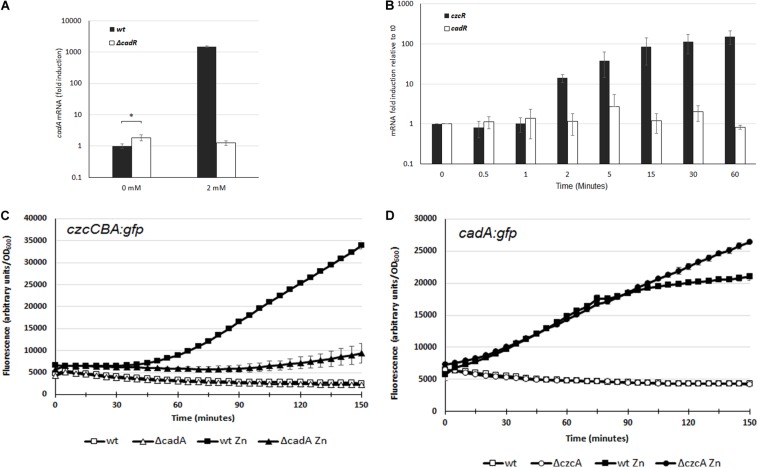
**(A)** Induction of *cadA* mRNA analyzed by qRT-PCR in the WT and the Δ*cadR* mutant cultivated in the absence (0 mM) or after 15 min of ZnCl_2_ (2 mM) as indicated. The amount of mRNA is represented relative to the WT strain cultivated in the absence of metal. Expression differences between WT and Δ*cadR* at 0 mM ZnCl_2_ are indicated using a *p*-value of <0.05 (*). **(B)** Fold induction of *czcR* and *cadR* mRNA analyzed using qRT-PCR on RNA extracted at various times following addition of 2 mM ZnCl_2_. Error bars represent the standard deviations of three independent determinations. **(C,D)** Fluorescence intensity measurement of *czcCBA::gfp* **(C)**, and *cadA::gfp* **(D)** in the WT, Δ*cadA* or Δ*czcC* in the absence of, or after the addition of 2 mM ZnCl_2_. The curve of the WT in the absence of Zn is masked behind the Δ*cadA* or Δ*czcC* curve. Values are normalized by optical density (OD_600_). Standard deviations (error bars) of three measurements are indicated.

In *P. aeruginosa*, the CadR/CadA system has primarily been studied in response to Cd excess whereas no characterization has been performed concerning its role in Zn resistance. The transcriptional activation of *cadA* is performed by the putative CadR transcriptional regulator (PA3689) located upstream of the *cadA* gene (PA3690), but transcribed in the opposite direction (Lee et al., 2001; Brocklehurst et al., 2003). To confirm that CadR is involved in the regulation of *cadA* expression also in a Zn excess situation, we constructed a deletion mutant for the *cadR* gene. The Zn MTC in the Δ*cadR* mutant was 6 mM, a concentration lower than the 8 mM of the WT, suggesting that the CadR regulator is also involved in Zn resistance (Table 1 and Supplementary Figure S1). The MTC of the Δ*cadR* mutant was higher than the Δ*cadA* mutant and this might reflect the basal transcription of the *cadA* gene, considering that CadR, in a similar fashion to the *E. coli* ZntR regulator, might be involved in the repression of CadA in absence of Zn (Brocklehurst et al., 1999). In order to test this hypothesis, we used qRT-PCR in order to quantify and compare the transcriptional level of *cadA* in the WT and Δ*cadR* mutant. In the WT we observed a 1000-fold induction after the addition of 2 mM Zn for 15 min (Figure 3A). This induction was absent in the Δ*cadR* mutant, confirming the role of the CadR activator in the Zn response. As postulated, the transcriptional repression mediated by CadR in the absence of Zn was also observed (Figure 3A).

To decipher the very fast induction of *cadA* transcription compared to *czcCBA* transcription, we quantified the expression of their respective transcriptional regulators. Using qRT-PCR analysis on RNA extracted at various times after addition of Zn, we observed that the amount of *cadR* mRNA is stable (Figure 3B). This suggests that the protein might be constitutively present in the cell or could be rapidly produced to activate the transcription of *cadA* upon Zn binding.

On the other hand, the amount of *czcR* mRNA increased after 2 min of Zn treatment, reaching a 150-fold induction after 1 h. It is known that CadA exports Zn from the cytoplasm to the periplasm, where the metal is detected by the sensor of the CzcRS TCS driving *czcCBA* transcription. By rapidly increasing Zn concentration in the periplasm, CadA might therefore be important for the activation of *czcCBA* operon transcription. To confirm our hypothesis, we used the *czcCBA*::*gfp* transcriptional fusion and followed the induction of fluorescence after Zn addition in a WT strain and in the Δ*cadA* mutant (Figure 3C). A very strong delay in the fluorescence intensity was measured in the absence of CadA, confirming the importance of this P-type ATPase for the strong and rapid activation of *czcCBA* genes expression.

If CadA is necessary for the full expression of CzcCBA, what happens in the opposite situation? To test whether *cadA* gene transcription is affected in the absence of the CzcCBA efflux pump, we transformed the WT strain and the Δ*czcA* mutant with the *cadA:gfp* fusion and followed the fluorescence induction after Zn addition (Figure 3D). The initial inductions, up to 70 min, were very similar between the two strains, suggesting that CzcCBA is not necessary for CadA expression. Interestingly, however, after 70 min, when *czcCBA* transcription started (Figure 3C), the transcription of *cadA* stopped increasing. This suggests that the efficient CzcCBA efflux pump takes over the initial CadA P-type ATPase activity. This model is strongly supported by our data showing a continuous increased in *cadA* gene transcription in a Δ*czcA* mutant background (Figure 3D).

Altogether, these results clearly demonstrate that the first response element of *P. aeruginosa* in the presence of Zn excess is the CadR/CadA system. This system may play the role of Zn sentinel, thus guaranteeing not only a rapid efflux of this metal outside the cytoplasm, but also a fast expression of the CzcCBA pump for a powerful Zn export into the extracellular medium. Importantly, our data highlight that these two different zinc export systems are not redundant but have complementary actions.

### Characterization of the *cadA* Promoter

**FIGURE 4:**
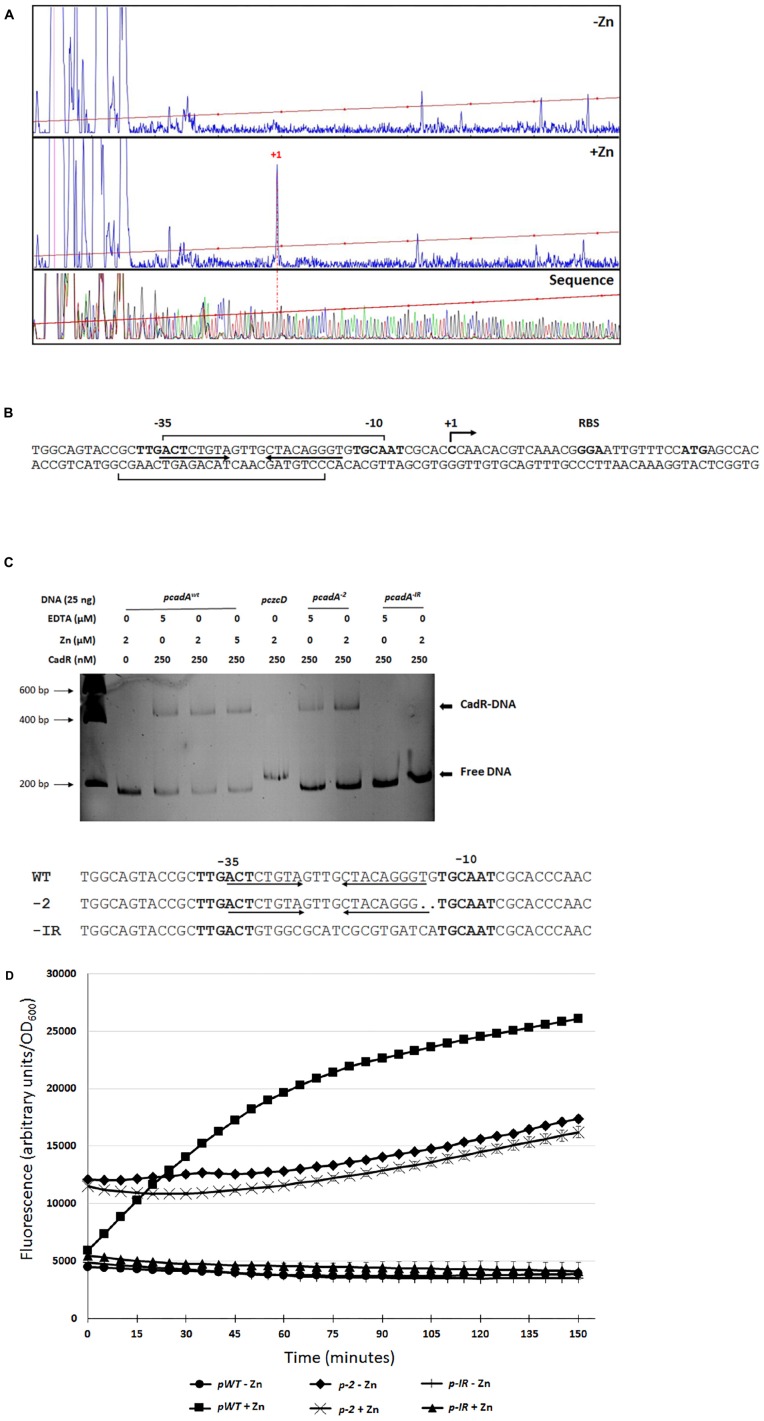
*cadA* promoter characterization. **(A)** Electropherogram of primer extension on the *cadA* transcript performed on RNA extracted from bacteria that have grown without (–Zn) or with (+Zn) zinc. The same FAM-labeled primer was used to perform the sequence analysis (Thermo Sequenase kit, Thermo Fisher Scientific). All reactions were analyzed by capillary electrophoresis (Microsynth AG, Switzerland). **(B)** Sequence of the *cadA* promoter, location of the transcriptional start site (+1), RBS (ribosome binding site) and ATG translational start codon. The –35 and –10 (relative to the +1 transcription start site) regions as well as the *cadR* box (inverted arrows), according to (Brocklehurst et al., 2003), are indicated. Results of the footprinting analysis are given using brackets on the coding and template strands (see Supplementary Figures S2A,B). **(C)** Electrophoretic mobility shift assay using purified CadR protein and *cadA* promoters. *pcadA*^xwt^ corresponds to the native promoter, the *czcD* promoter (*pczcD*) is used as a negative control. *pcadA^–2^* and *pcadA^–IR^* correspond the *cadA* WT promoter with a deletion of two nucleotides and to the promoter without the *cadR* box, respectively. Sequences of the three promoters are indicated. **(D)** Transcriptional fusion of the different *cadA* promoters to the | *gfp* gene. Measurements were performed in triplicate in the presence or absence of Zn, as indicated.

We decided to characterize the regulation of *cadA* gene expression in the presence of Zn by analyzing its promoter. Primer extension analysis revealed a transcription start site at −27 nucleotides upstream the *cadA* ATG translation start codon (Figures 4A,B). We then purified the *P. aeruginosa* CadR protein (Supplementary Figure S2) and performed DNAse I footprinting analysis, confirming the location of CadR between the −35 and −10 region (Figure 4B and Supplementary Figures S3A,B) previously determined *in silico* (Brocklehurst et al., 2003).

CadR is a member of the MerR-type family of regulators that are known to bind the promoter in the absence of metal ligand (Outten et al., 1999). To confirm that CadR is binding to the DNA even in the absence of Zn, we performed an electrophoretic mobility shift assay (EMSA) using the *cadA* promoter and the purified CadR protein (Figure 4C). A shift was clearly visible in the presence of CadR, even in the absence of Zn or in addition of EDTA to chelate any trace metals. This shift could not be observed using another DNA promoter (*pczcD*) or when the *cadR* box was mutated (*pcadA^–IR^*). As observed in *E. coli* (Brocklehurst et al., 1999), the removal of two nucleotides in the region between the −35 and the −10 (*pcadA*^–2^) did not affect the binding of CadR (Figure 4C) and allowed constitutive *cadA* expression, even in the absence of Zn as measured by GFP promoter fusions (Figure 4D).

### Metal Specificity of the CadR/CadA System in *P. aeruginosa*

In order to better characterize the specificity of the CadA/CadR system in *P. aeruginosa*, we tested the resistance profile of the CadR and CadA mutant to several metals (Table 2). The MTC data showed that this system is involved not only in resistance to Zn and Cd, but also weakly to Pb. In contrast, it does not appear to be involved in Co, Ni, or Cu resistance. Complementation of the *cadR* mutant using a plasmid carrying a WT copy of the *cadR* or *cadA* gene restored the metal tolerance of the mutants to the wild-type levels, showing that no polar effect was caused by the deletion.

### Induction of Metal Resistance During Phagocytosis

**FIGURE 5:**
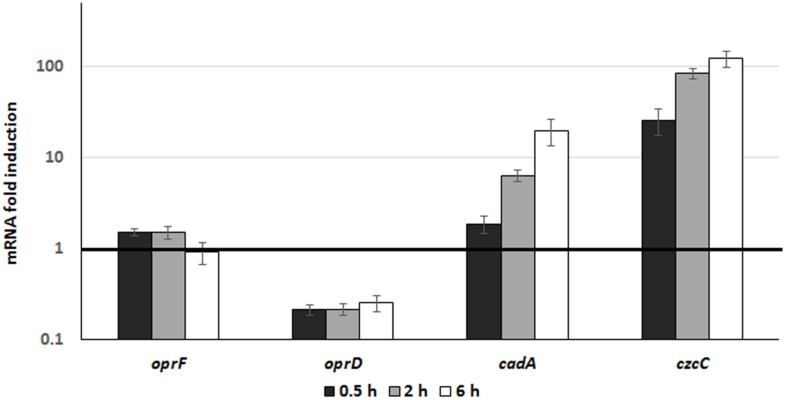
Gene expression analysis during phagocytosis. *P. aeruginosa* WT strain was used to infect THP1 macrophages (MOI 2.5) for 1 h. RNA from culture medium, corresponding to unphagocytosed bacteria, was extracted and used for comparison (dark line set to 1 on the figure). RNA from macrophages was then extracted 0.5, 2, and 6 h post-infection. Mean values and standard deviations (error bars) of three independent experiments are indicated.

In order to monitor the involvement and induction of CadA and CzcCBA during phagocytosis, we followed the expression of the *czcC* (representing the *czcCBA* operon) and *cadA* genes once *P. aeruginosa* were inside macrophages. To this end, we used differentiated THP-1 cells and extracted RNA at 30 min, 2 and 6 h after infection with *P. aeruginosa*. Gene expression inside the macrophage was compared to expression in free-living bacteria (medium containing un-phagocytosed bacteria). Using qRT-PCR, we observed a time-dependent induction of *czcC* and *cadA* mRNA. After 6h, the expression of *cadA* increased 20-fold and *czcC* more than 100-fold (Figure 5). This suggests that *P. aeruginosa* respond to phagocytosis by the induction of these Zn resistance efflux mechanisms. However, in these conditions no differences in induction kinetics could be observed in contrast to *in vitro* measurements in an Erlenmeyer flask (Figure 2). Importantly, while expression of the general porin *oprF* was not affected, we observed a rapid decrease in *oprD* mRNA levels. This repression is known to lead to carbapenem resistance and be mediated by the CzcRS TCS in response to Zn or Cu excess (Perron et al., 2004; Caille et al., 2007).

## Discussion

*Pseudomonas aeruginosa* is a highly versatile bacterium capable of living in heavy metal-contaminated environments, but also of reacting to metals by enhancing its virulence and antibiotic resistance (Gonzalez et al., 2019). In this study, we focused on the export systems involved in resistance to Zn and we found that the two major systems are the CadA P-type ATPase and the CzcCBA efflux pump. According to our results on their expression dynamics, *cadA* is very rapidly expressed in response to Zn excess and its expression remains maximal until CzcCBA takes over.

**FIGURE 6:**
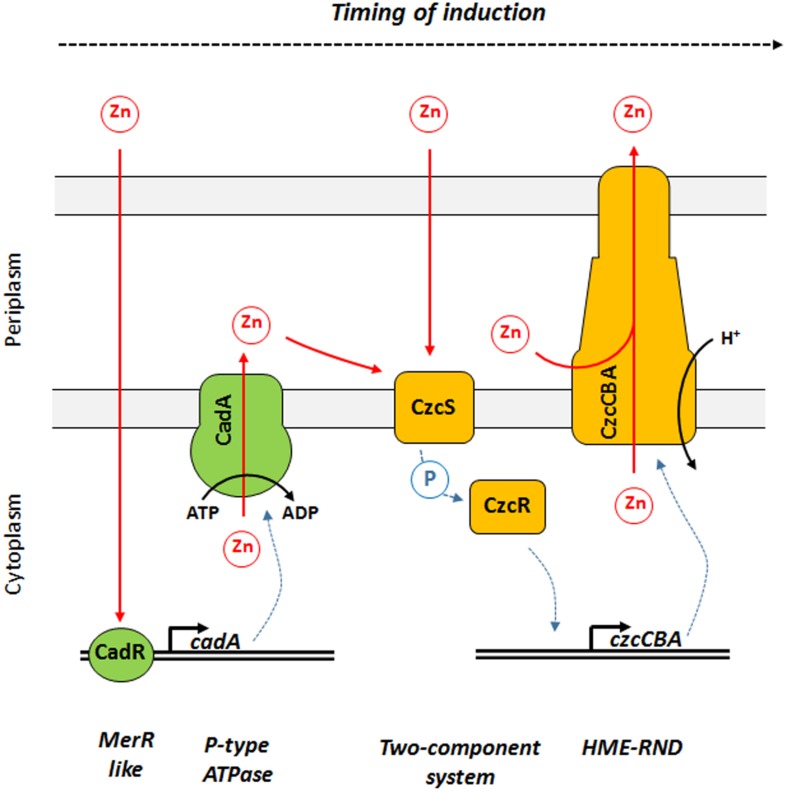
Model of Zn response dynamics in *P. aeruginosa*. From left to right: once Zn is in the cytoplasm it might be directly detected by the MerR-like regulator CadR. This protein is already present on the DNA and activates the transcription of *cadA*, encoding a P-Type ATPase capable of rapidly expelling Zn from the cytoplasm to the periplasm using ATP hydrolysis as energy source. Zn in the periplasm might then be detected by CzcS, the sensor protein of the CzcRS two-component system. Upon dimerization, CzcS activate the cytoplasmic CzcR response regulator by phosphorylation. CzcR will then activate its own transcription (not represented in this diagram) as well as the transcription of the *czcCBA* efflux pump. CzcCBA is part of the HME RND efflux pump that is capable of expelling Zn from the periplasm and/or cytoplasm directly outside the bacterium using proton motive force.

In this study, we showed that *P. aeruginosa* CadA/CadR is the first system to react to Zn excess and is playing the role of sentinel of Zn concentrations (Figure 6). We propose a model in which the transcriptional regulator CadR is already present on the *cadA* promoter and, upon Zn binding, is rapidly inducing the transcription of this P-type ATPase. CadA is then capable of removing toxic concentrations of cytoplasmic Zn by expelling it into the periplasm. The Zn excess in the periplasm might then be detected by CzcS, the sensor of the CzcRS TCS that in turn activates CzcR by phosphorylation and drives the transcription of the *czcCBA* efflux pump. This RND machinery is capable of expelling Zn directly outside the bacterium either from the periplasm, from the cytoplasm or both (Nies, 2003; Figure 6).

We show here that the two efflux systems are not redundant but rather complementary; with CadA/CadR providing a rapid reaction to bring Zn into the periplasm, bursting the expression of *czcCBA* in order to expel the Zn outside the cell. Indeed, although CadA/CadR is the first system to react to Zn, the CzcCBA efflux pump is more efficient according to the MTC of the mutants (Table 1); a *cadA*-deficient mutant can grow in presence of up to 5 mM Zn, while the *czcA* mutant only in 3 mM. This CzcCBA system has already been described as the first layer of metal resistance (Nies, 2003). Interestingly, in the presence of Zn the growth of the *czcA/cadA* double mutant was more strongly affected than single mutants, which clearly underlines the synergy in metal resistance between CadA and CzcCBA export systems. This functional interaction between CadA and CzcCBA could also exist between other export systems. Even though we did not observe any effect of CDFs on Zn resistance under our conditions, we cannot exclude that their action would be significant in the absence of CadA or CzcCBA.

Pathogens can use bacterial Zn export systems to thrive under conditions of high metal contamination (Nies, 2003; Chandrangsu et al., 2017), but also to counteract the toxic metal concentrations discharged into the phagolysosome after phagocytosis (Botella et al., 2012; Djoko et al., 2015; Hao et al., 2016). Using qRT-PCR, we observed an induction of *czcCBA* and *cadA* genes in *P. aeruginosa* after phagocytosis by THP1-differentiated macrophages. A similar 20-fold induction of *zntA* was recently observed in *E. coli* phagocytosed by THP1 cells (Kapetanovic et al., 2016). We were unable, however, to detect the rapid subsequent expression of *cadA* and *czcCBA* inside macrophages, probably due to the non-synchronized phagocytosis events rendering early measurement not feasible. Moreover, the two situations (phagocytosis versus addition of Zn to the culture medium) are not comparable and the lower fold induction in the macrophage (Figure 5) compared to the culture medium containing 2 mM Zn (Figure 2A), may mask differences.

In the future, we plan to further investigate this particular aspect by looking at single-cells events, as it will be important to understand how the pathogen respond to and resist the discharge of metals such as Zn or Cu that occur in a phagolysosome (Djoko et al., 2015).

Importantly, qRT-PCR analysis clearly demonstrated that *oprD*, encoding a porin involved in the entry of small peptides and carbapenem antibiotics (Trias and Nikaido, 1990a, b), is repressed within macrophages (Figure 5). It is known that the induction of CzcR in the presence of Zn excess (Perron et al., 2004) is able to repress *oprD* porin expression, leading to carbapenem resistance. Most importantly, our data suggest that macrophages could represent a reservoir for selection of carbapenem resistant strains. We have previously shown that high Zn concentrations are capable of selecting *P. aeruginosa* strains resistant to carbapenems even in the absence of these antibiotics (Perron et al., 2004) and several carbapenem-resistant mutants, arising from mutations in the *czcRS* genes, have been isolated from patients and animals (Fournier et al., 2013; Haenni et al., 2017).

Finally, the analysis of the *P. aeruginosa cadA* promoter allows us to confirm the location of the *cadR* box between the −35 and −10 regions, as suggested by previous analysis (Brocklehurst et al., 2003) and corresponding to a clear signature of a *cadR* box (Permina et al., 2006). We show here that the CadA P-type ATPase confers resistance not only to Cd and Zn, but also to Pb. In addition to CzcCBA, also leading to Zn, Cd and Co resistance, these two systems might be important for *P. aeruginosa* to grow in metal-contaminated environments in addition to the infectious processes. The analysis of the expression dynamics of these metal resistance systems could be of prime relevance to understanding the behavior and adaptability of *P. aeruginosa* in metal-enriched situations.

## Data Availability Statement

All datasets generated for this study are included in the article/Supplementary Material.

## Author Contributions

VD and KP designed the study. VD, SL, and MG performed the experiments. VD, MV, and KP analyzed the data. All authors discussed the results, wrote the manuscript, and approved the final version of the manuscript.

## Conflict of Interest

The authors declare that the research was conducted in the absence of any commercial or financial relationships that could be construed as a potential conflict of interest.
