# Supplementary figures and images for: The CzcCBA Efflux System Requires the CadA P-Type ATPase for Timely Expression Upon Zinc Excess in Pseudomonas aeruginosa
Source: Front Microbiol. 2020 May 15;11:911. doi: 10.3389/fmicb.2020.00911 (PMC7242495; doi:10.3389/fmicb.2020.00911)

**Figure S1:** Representative image of CMT analysis.

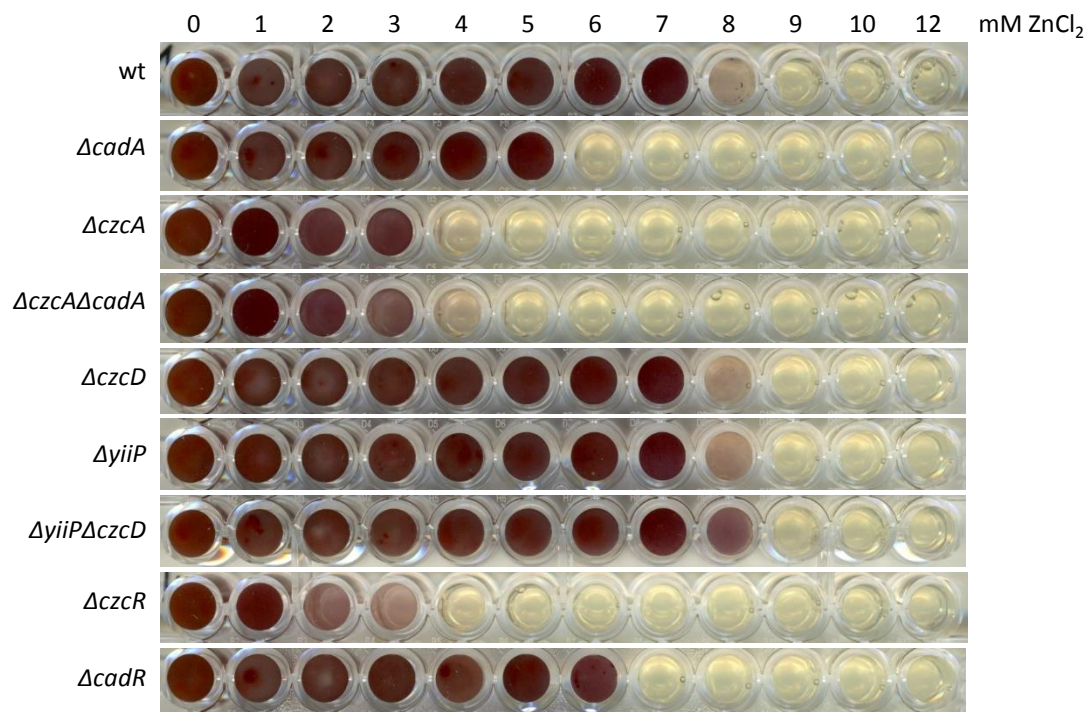

Supplement: Supplementary file 1 [file Data_Sheet_1.PDF]
