# Supplementary material for: The CzcCBA Efflux System Requires the CadA P-Type ATPase for Timely Expression Upon Zinc Excess in Pseudomonas aeruginosa
Source: Front Microbiol. 2020 May 15;11:911. doi: 10.3389/fmicb.2020.00911 (PMC7242495; doi:10.3389/fmicb.2020.00911)

Figure S2: CadR SDS-PAGE. 2 µl of a 25 µM (0.9 µg) of purified CadR protein was loaded onto a 4-12% electrophoresis gel and stained with Coomassie blue.

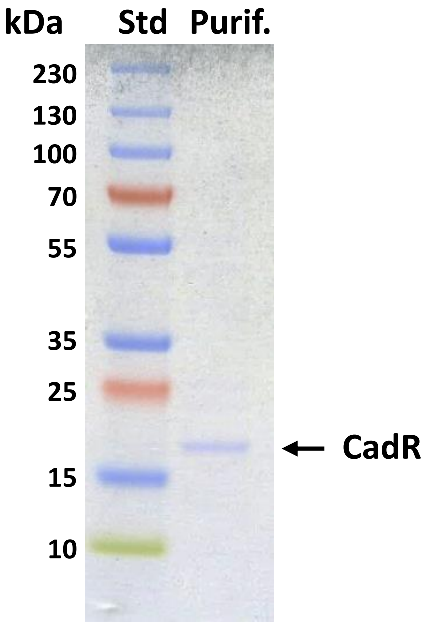

Supplement: Supplementary file 2 [file Data_Sheet_2.PDF]
