# Supplementary material for: The CzcCBA Efflux System Requires the CadA P-Type ATPase for Timely Expression Upon Zinc Excess in Pseudomonas aeruginosa
Source: Front Microbiol. 2020 May 15;11:911. doi: 10.3389/fmicb.2020.00911 (PMC7242495; doi:10.3389/fmicb.2020.00911)

Figure S3: CadR DNAse I footprinting of *cadA* promoter. **(A)** Coding strand. **(B)** Template strand. The DNAse I reaction on the FAM-labeled promoters was performed in the presence or absence of CadR protein, as indicated. DNA fragments were analyzed by capillary electrophoresis. The sequencing reaction was performed using the same FAM-labeled primers and analyzed in the same capillary electrophoresis. All peaks were analyzed using PeakScanner2 software (Thermofisher Scientific).

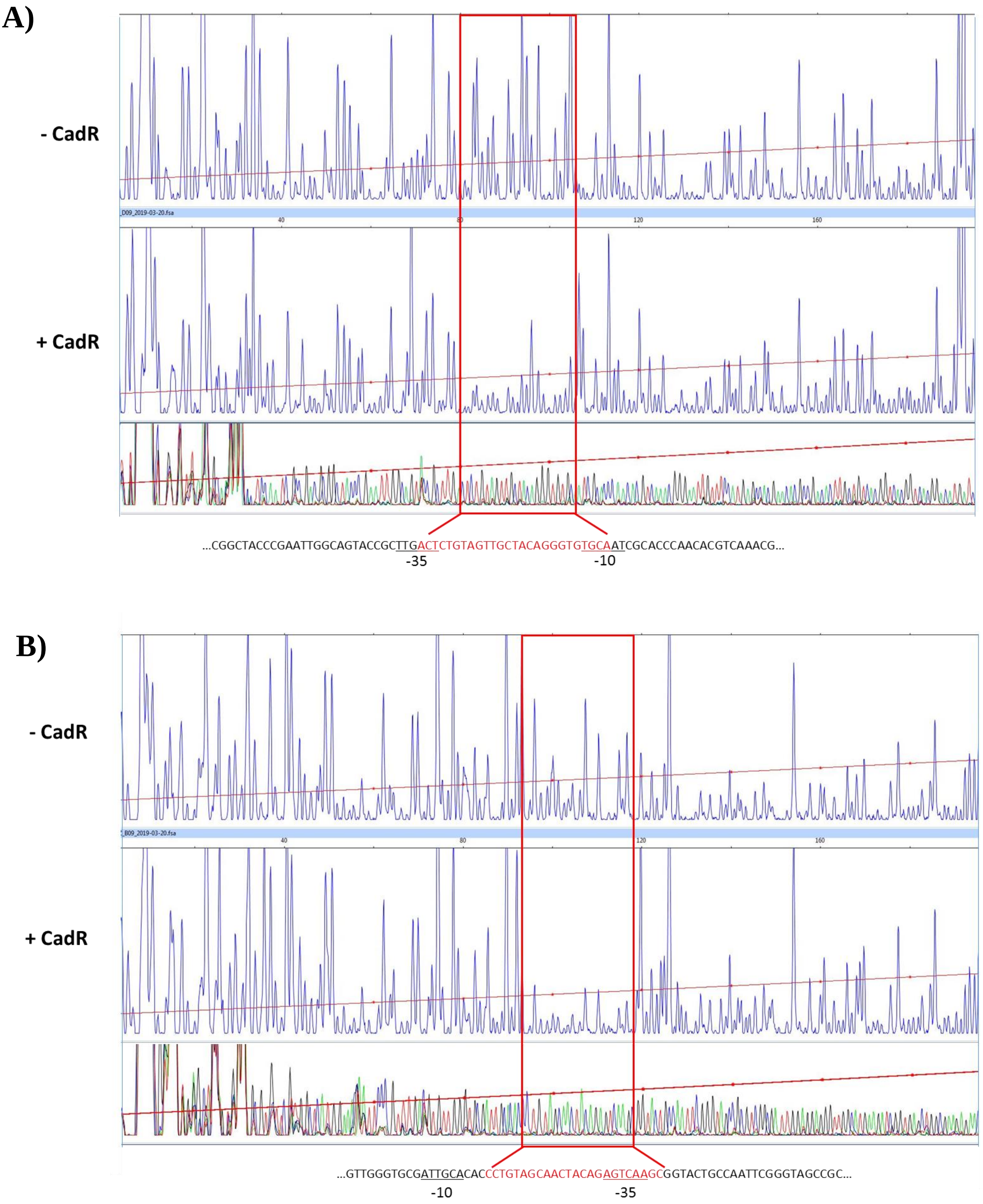

Supplement: Supplementary file 3 [file Data_Sheet_3.PDF]
