# Supplementary material for: The CzcCBA Efflux System Requires the CadA P-Type ATPase for Timely Expression Upon Zinc Excess in Pseudomonas aeruginosa
Source: Front Microbiol. 2020 May 15;11:911. doi: 10.3389/fmicb.2020.00911 (PMC7242495; doi:10.3389/fmicb.2020.00911)
Supplement: Supplementary file 4 [file Data_Sheet_4.PDF]

Table S1: Strains and plasmids used in this study

Strain or plasmid	Relevant characteristic(s)[a]	Reference/source
P. aeruginosa		
Wild type	PAO1 wild type	Laboratory collection
ΔczcA	PAO1 Δ*czcA*	(Perron et al., 2004)
ΔcadA	PAO1 Δ*cadA*	This study
ΔczcAΔcadA	PAO1 Δ*czcA*Δ*cadA*	This study
ΔczcD	PAO1 Δ*czcD*	This study
ΔyiiP	PAO1 Δ*yiiP*	This study
ΔczcDΔyiiP	PAO1 Δ*czcD*Δ*yiiP*	This study
ΔczcR	PAO1 Δ*czcR*	This study
ΔcadR	PAO1 Δ*cadR*	This study
PT1108	PAO1 carrying the constitutive CzcS(V194L) mutation	(Perron et al., 2004)
E. coli		
DH5α	recA1, endA1, hsdR17, deoR, thi-1, supE44, gyrA96, relA1, Δ(lacZYA-argF), U169(φ80dlacZΔM15)	(Sambrook, 2001.)
BL21(DE3)	E. coli str. B F– ompT gal dcm lon hsdSB(rB–mB–) λ(DE3 [lacI lacUV5-T7p07 ind1 sam7 nin5]) [malB+]K-12(λS)	(Studier et al., 1990)
**Plasmids**		
pME3087	Suicide plasmid, Co1E1 replicon; Tc^r	(Wenner et al., 2014)
pME6001	pME6000 derivative plasmid; Gm^r	(Blumer et al., 1999)
pME6001-*cadA*	pME6000 derivative , carrying the *zntA* gene under its own promoter; Gm^r	This study
pME6001-*cadR*	pME6000 derivative , carrying the *zntR* gene under its own promoter; Gm^r	This study
pBBR1-*gfp*	Transcriptional *gfp* fusion cloning vector; Ap^r, Cb^r	(Ouahrani-Bettache et al., 1999)
czcCBA ::gfp	pBBR1 derivative , carrying the *czcCBA* promoter; Ap^r, Cb^r	This study
cadA ::gfp/ pWT	pBBR1 derivative , carrying the *cadA* wild type promoter; Ap^r, Cb^r	This study
p-2	pBBR1 derivative, carrying the *cadA* promoter with a 2 nucleotides deletion in the CadR box; Ap^r, Cb^r	This study
p-IR	pBBR1 derivative, carrying the *cadA* promoter deleted for the CadR box; Ap^r, Cb^r	This study
pGex-2T-*cadR*	GST-fusion expression plasmid	This study
[a]Antibiotic resistance are indicated by r: Ap, ampicillin, Gm, gentamicin; Tc, tetracycline; Cb, carbenicillin.		

References:

Blumer, C., Heeb, S., Pessi, G., and Haas, D. (1999). Global GacA-steered control of cyanide and exoprotease production in Pseudomonas fluorescens involves specific ribosome binding sites. *Proc Natl Acad Sci U S A* 96(24)**,** 14073-14078. doi: 10.1073/pnas.96.24.14073.

Ouahrani-Bettache, S., Porte, F., Teyssier, J., Liautard, J.P., and Kohler, S. (1999). pBBR1-GFP: a broad-host-range vector for prokaryotic promoter studies. *Biotechniques* 26(4)**,** 620-622. doi: 10.2144/99264bm05.

Perron, K., Caille, O., Rossier, C., Van Delden, C., Dumas, J.L., and Kohler, T. (2004). CzcR-CzcS, a two-component system involved in heavy metal and carbapenem resistance in Pseudomonas aeruginosa. *J Biol Chem* 279(10)**,** 8761-8768. doi: 10.1074/jbc.M312080200

M312080200 [pii].

Sambrook, J., and D. W. Russell. (2001.). *Molecular cloning: a laboratory manual* 3rd ed.Cold Spring Harbor Laboratory Press, Cold Spring Harbor, NY.

Studier, F.W., Rosenberg, A.H., Dunn, J.J., and Dubendorff, J.W. (1990). Use of T7 RNA polymerase to direct expression of cloned genes. *Methods Enzymol* 185**,** 60-89.

Wenner, N., Maes, A., Cotado-Sampayo, M., and Lapouge, K. (2014). NrsZ: a novel, processed, nitrogen-dependent, small non-coding RNA that regulates Pseudomonas aeruginosa PAO1 virulence. *Environ Microbiol* 16(4)**,** 1053-1068. doi: 10.1111/1462-2920.12272.
