# Supplementary material for: The CzcCBA Efflux System Requires the CadA P-Type ATPase for Timely Expression Upon Zinc Excess in Pseudomonas aeruginosa
Source: Front Microbiol. 2020 May 15;11:911. doi: 10.3389/fmicb.2020.00911 (PMC7242495; doi:10.3389/fmicb.2020.00911)
Supplement: Supplementary file 5 [file Data_Sheet_5.PDF]

**Table S2:** Primers used in this study

| Amplicon | Primer | Sequence 5'-3' | Position | Length |
|---|---|---|---|---|
| | | | | |
| **Coding regions** | | | | |
| *czcC* | czcC1 | GGTCAGCATCGGCAGCAAGTACG | 834 | 206 |
| | czcC2 | GGTCGTAGGCCTGTACCGCTTCG | 1039 | |
| | | | | |
| *cadA* | 1086 | CATCAACGCCCTGATGAGTA | 543 | 231 |
| | 1087 | GCTTCCAGTTCCACTTGCTT | 773 | |
| | | | | |
| *czcR* | 1129 | GGAGACCATCCGCTACTACG | 42 | 160 |
| | 1130 | CAGCAGGGTACGGATTTCCT | 201 | |
| | | | | |
| *cadR* | czcR1 | GTCATCACCCGGACGCAGATCAT | 502 | 153 |
| | czcR2 | GTAGCCGACGCCGCGAATGGTAT | 654 | |
| | | | | |
| *oprD* | oprD1 | ATCTACCGCACAAACGATGAAGG | 772 | 156 |
| | oprD2 | GCCGAAGCCGATATAATCAAACG | 927 | |
| | | | | |
| *oprF* | oprF1 | GGTTACTTCCTGACCGACGA | 172 | 209 |
| | oprF2 | TCGCTGTTGATGTTGGTGAT | 380 | |
| | | | | |
| *rpoS* | 497 | TTTGACCACGATGATGAAGT | 27 | 185 |
| | 498 | CGATTTCGTTGAGATACAGC | 211 | |
| | | | | |
| **Promoter regions** | | | | |
| *pcadA* | 1111 | AACCGGTTCTCTTCGCCAGC | -147 | 205 |
| | 1112 | CCATGATCATGTCCGTGACC | 58 | |
| | | | | |
| *pczcD* | 1101 | TCGTGCTGTTGAGTCATGCG | -168 | 247 |
| | 1102 | GCTTCCGGTCAGCAGCAACG | 77 | |
| | | | | |
| *pcadA* | 1196 | 6FAM-AACCGGTTCTCTTCGCCAGC | -147 | |
| | 1197 | 6FAM-CCATGATCATGTCCGTGACC | 58 | |
| | | | | |
| **DNA clonings** | | | | |
| *ΔcadA* | 1149 | ATTCGAGCTCGGTACCCGGGGCTCGGTCGGCGGGTTGC | 2723 | 501 |
| | 1150 | CCGAGGAACGAAGGCCCCACTGGCCTCC | 2223 | |
| | 1151 | GTGGGGCCTTCGTTCCTCGGTCGGGCAG | 172 | 579 |
| | 1152 | CCTGCAGGTCGACTCTAGAGAGTTCGGCGATGCGCACC | -406 | |
| | | | | |
| *ΔcadR* | 1137 | ATTCGAGCTCGGTACCCGGGGGCCAGCGGCCACCAGGG | -500 | 540 |
| | 1138 | TGCATGTGCCCCGGGCAACCGGTTCTCTTCGC | 39 | |
| | 1139 | GGTTGCCCGGGGCACATGCACGTCCCCG | 430 | 390 |
| | 1140 | CCTGCAGGTCGACTCTAGAGAGATCCGTGAGCTTGTCCGC | 819 | |
| | | | | |
| *ΔczcR* | 896 | ATTCGAGCTCGGTACCCGGGCCACGCAACCGTTCATCG | -454 | 453 |

| | | | | |
|---|---|---|---|---|
| | 897 | GTGGCGTCATGTTCGCCCCTATATAAAGTATGG | -2 | |
| | 898 | AGGGGCGAACATGACGCCACCAGGCCGA | 671 | 511 |
| | 899 | CCTGCAGGTCGACTCTAGAGAACCGTCGAGCAGGCGCT | 1181 | |
| | | | | |
| *ΔczcD* | 584 | GCCGAATTCGGTGAACAGGTAGATGATCC | -471 | 440 |
| | 585b | GCCGGTACCCAGCCTACAGGATAGGCAAT | -32 | |
| | 586b | GCCGGTACCAAGACGGTTCAGCGGAAGCG | 899 | 440 |
| | 587 | GCCAAGCTTATCATCCACTTCTTCCCGGC | 1338 | |
| | | | | |
| *ΔyiiP* | 1190 | ATTCGAGCTCGGTACCCGGGCCTTGCCACCCGCGTTTCCTGTG | -500 | 531 |
| | 1191 | GGTGCAACTGCAGGCGGGCGTGGGGGGA | 30 | |
| | 1192 | CGCCCGCCTGCAGTTGCACCTCGAACTGC | 744 | 556 |
| | 1193 | CCTGCAGGTCGACTCTAGAGCCGCGCTATGTGCTGGTC | 1299 | |
| | | | | |
| *cadA:gfp* | 1198 | CGCGGTACCAACCGGTTCTCTTCGCCAGC | -168 | 246 |
| | 1199 | GCCAGATCTCCATGATCATGTCCGTGACC | 77 | |
| | | | | |
| *czcC:gfp* | 688 | GCCGGTACCGGCGTGAGGGGCAATGCCTT | -257 | 302 |
| | 689 | GCCAGATCTGTTCCGCTCCTCGTCTGCTG | 44 | |
| | | | | |
| pME6001:*cadA* | 1205 | GCCAAGCTTGCCAACCCTCCTCCAATCGCC | -113 | 2354 |
| | 1206 | GCCGGATCCGGAGGCCAGTGGGGCCTT | 2240 | |
| | | | | |
| pME6001:*cadR* | 1188 | GCCAAGCTTTCCATCGCTTCGATGCGCAG | -268 | 751 |
| | 1189 | GCCGGATCCCTGAGCGCGGCTTCAGCCAT | 482 | |
| | | | | |
| pGex2T:*cadR* | 1182 | CGGGGATCCATGAAGATCGGTGAGCTGGC | 1 | 470 |
| | 1185 | GCGGGATCCTCAGCCATGCCGGCGGTGCA | 470 | |
