# Supplementary material for: The CzcCBA Efflux System Requires the CadA P-Type ATPase for Timely Expression Upon Zinc Excess in Pseudomonas aeruginosa
Source: Front Microbiol. 2020 May 15;11:911. doi: 10.3389/fmicb.2020.00911 (PMC7242495; doi:10.3389/fmicb.2020.00911)
Supplement: Supplementary file 6 [file Data_Sheet_6.PDF]

Table S3: Metal concentrations (mM) used for the experiment presented in Table 3

**$ZnCl_2$**	0	2	3	4	5	6	8	10
**$CdCl_2$**	0	0.25	1	2	3	4	5	6
**$CoCl_2$**	0	0.25	0.5	1	1.5	2	2.5	3
**$NiCl_2$**	0	1.5	2	2.5	3	3.5	4	4.5
**$CuCl_2$**	0	1	2	3	4	5	6	7
**$Pb(NO_3)_2$**	0	2	4	6	8	10	12	14
